# Computed tomography-guided resection of osteoid osteoma of the sacrum: a case report

**DOI:** 10.1186/1752-1947-8-206

**Published:** 2014-06-18

**Authors:** Shinsuke Fukuda, Michiro Susa, Itsuo Watanabe, Kazumasa Nishimoto, Keisuke Horiuchi, Yoshiaki Toyama, Hideo Morioka

**Affiliations:** 1 Department of Orthopedic Surgery, School of Medicine, Keio University, 35 Shinanomachi, Shinjuku-ku, Tokyo 160-8582, Japan

**Keywords:** Osteoid osteoma, Sacrum, CT-guided resection

## Abstract

**Introduction:**

Osteoid osteoma is a benign tumor that usually occurs in the long bones of young adults. Its symptoms can be diverse depending on the location of the tumor and especially difficult to diagnose when occurring in an atypical location. Osteoid osteoma arising in the sacrum is extremely rare, and here, we present a case that was treated successfully in a minimally invasive fashion under computed tomography guidance.

**Case presentation:**

A 25-year-old Asian man was referred to our institution due to persistent pain in the buttock after 12 months of conservative treatment. Computed tomography and magnetic resonance imaging scans revealed a ring-shaped radiolucency consistent with a nidus of osteoid osteoma in the sacrum. The lesion was subsequently resected under computed tomography guidance and the histological diagnosis was compatible with osteoid osteoma. His postoperative course was uneventful, and at two years after surgery our patient is symptom-free with no evidence of recurrence.

**Conclusions:**

Computed tomography-guided resection of osteoid osteoma in the spinal column is feasible and accurate if there is adequate margin from vital organs. Although rare, it is important to always bear in mind the possibility of osteoid osteoma occurring in the sacrum when no other apparent lesion is detected.

## Introduction

Osteoid osteoma is a benign tumor of the bone that occurs predominantly in the lower extremity in young adults. The tumor can arise in any part of the skeletal tissue; however, it is rarely found in the spine. The lesion is commonly characterized as a small calcified lesion (<2cm) on X-rays with disproportionate pain that aggravates at night. Usually, the nocturnal pain is effectively alleviated by the administration of nonsteroidal anti-inflammatory drugs. The tumor sometimes causes pain even before characteristic findings on X-rays are discernible and is commonly associated with referred pain in a nearby joint. Because of these obscurities of osteoid osteoma, in addition to its relatively low occurrence, the correct diagnosis is often very challenging. In this report, we present a rare case of osteoid osteoma arising in the sacrum that was successfully treated by computed tomography (CT)-guided resection, with a review of the cases of osteoid osteoma arising in the sacrum.

## Case presentation

**Figure 1:**
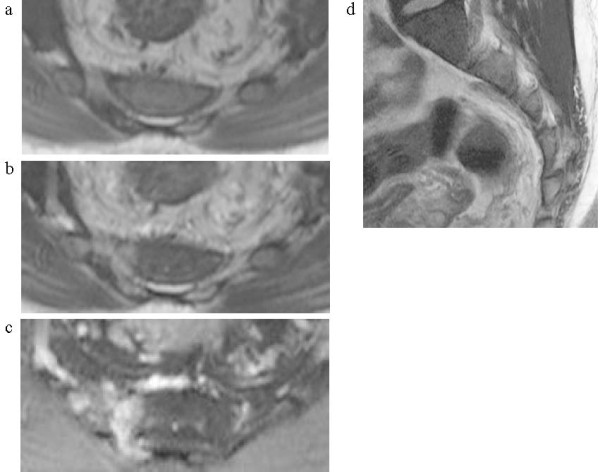
**Magnetic resonance imaging scan of the sacrum.** On the magnetic resonance imaging scan, the tumor was depicted as a low-intensity lesion on T1-weighted images **(a)** and high-intensity lesion on both T2-weighted images **(b)** and T2-weighted selective partial inversion recovery images **(c, d)**.

**Figure 2:**
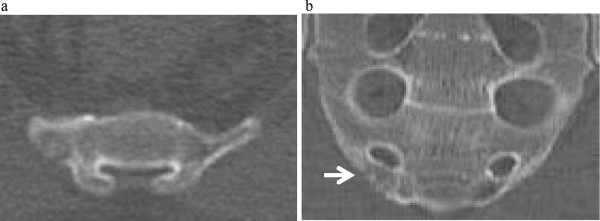
**Computed tomography scan of the sacrum.** The computed tomography scan shows a 9×5mm osteolytic lesion with minute mineralization in the lower end of right ala of the sacrum (arrow). **(a)** Axial image. **(b)** Coronal image.

**Figure 3:**
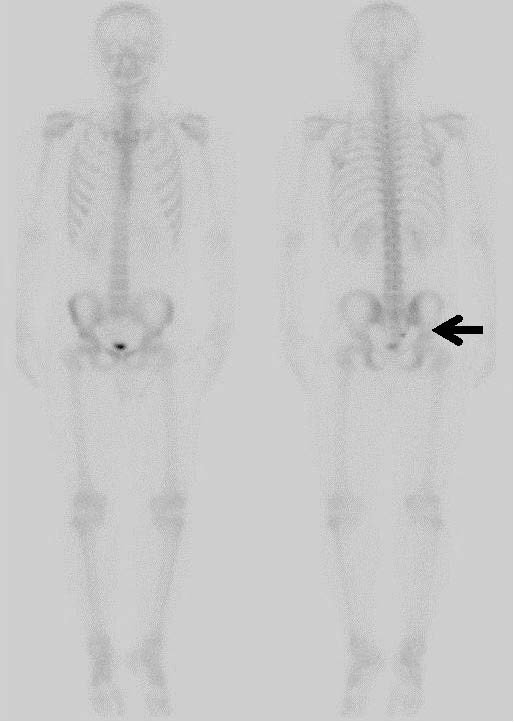
**Bone scintigraphy.** The tumor is depicted as an uptake in the right lower end of the sacrum (arrow).

**Figure 4:**
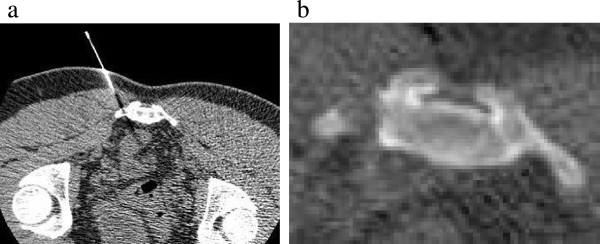
**Computed tomography-guided resection. (a)** A guide pin has been inserted into the nidus under computed tomography guidance. **(b)** Postoperative computed tomography shows a successful resection of the nidus.

**Figure 5:**
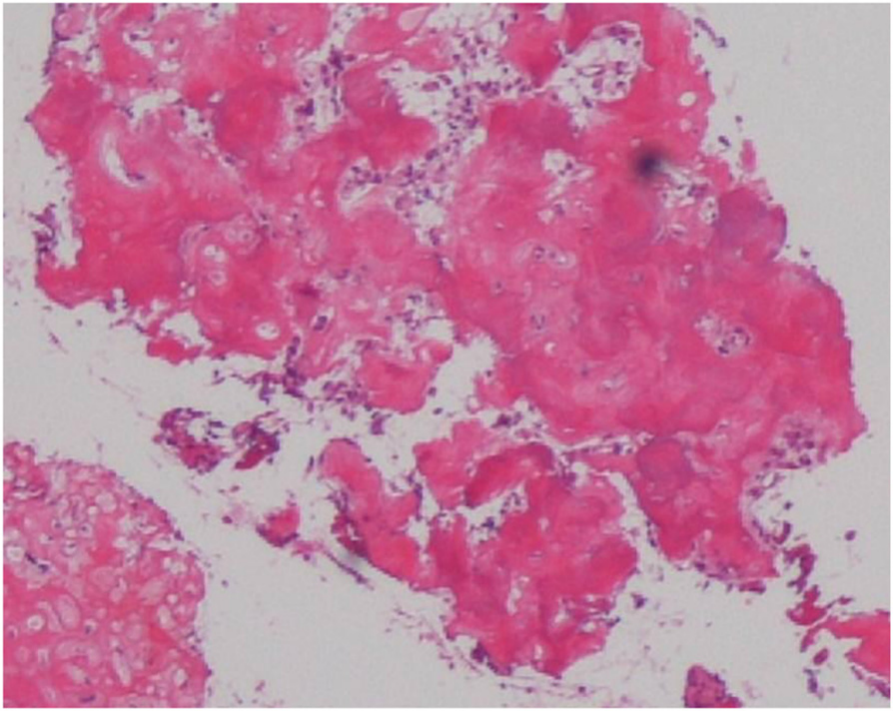
**Histological examination of the specimen.** Photomicrograph of hematoxylin and eosin staining shows an osteogenic tumor composed from interconnected trabeculae of woven bone and differentiated osteoblasts lining the osteoid, consistent with osteoid osteoma. Original magnification ×200.

A 25-year-old Asian man complained of a persistent pain in the buttock, which worsened at night. He went to a nearby hospital; however, neither physical examination nor X-rays showed apparent abnormalities. A nonsteroidal anti-inflammatory drug was prescribed for a pain of unknown cause. The medication was quite effective, but after a year of prescription, the pain gradually deteriorated and our patient began to experience night pain that interfered with sleep. Because the origin of the pain was still unknown, he was referred to our institute for further examination. Our patient had no previous incidence of trauma or any relevant family or medical history. On physical examination, there was no redness or swelling, but there was tenderness on the right lower sacrum. The overlying skin was smooth and nonadherent, and there was no neurological deficit. On X-ray, no abnormal finding was detected. On the magnetic resonance imaging (MRI) scan, there was an approximately 1cm lesion in the S4 ala of sacrum that extended into the posterior soft tissue, which showed low intensity on T1-weighted images and high intensity on both T2-weighted images and T2-weighted selective partial inversion recovery (SPIR) images with unclear margin, indicative of bone edema or inflammation (Figure 1). A CT scan was performed twice. The first CT scan showed no abnormality, but the clinical symptoms and the MRI scan strongly suggested an osteoid osteoma A repeat CT scan was performed, this time with thin-slice CT. There was a 9×5mm osteolytic lesion in the lower end of the right ala of the sacrum with minute mineralization inside the lesion, which was consistent with the characteristic ring-shaped radiolucency (nidus) found in osteoid osteoma (Figure 2). In accordance, bone scintigraphy revealed an uptake in the same location where a lesion was detected on CT and MRI scans (Figure 3). Based on these clinical and radiological findings, a diagnosis of osteoid osteoma of the sacrum was finally made, and a CT-guided resection was planned for the treatment. The operative procedure was performed in the CT room under general anesthesia. After identifying the nidus with a lead marker, the direction and depth of guide pin insertion was determined and a guide pin was inserted by a power drill just adjacent to the edge of the nidus. A 5.0mm cannulated drill was inserted over the guide pin to remove the lesion and the bone specimen that resided in the cannulated drill was sent for histological study. Heat ablation was subsequently performed by electrosurgical knife using a standard electrosurgical generator. The duration of the operation was one hour and six minutes with minimal hemorrhage (Figure 4). Histologically, hematoxylin and eosin staining demonstrated differentiated osteoblasts lining the osteoid, and interconnected trabeculae of woven bone, which was compatible with osteoid osteoma (Figure 5). The postoperative period was uneventful, and at the final follow-up visit after two years, no local recurrence was observed.

## Discussion

Osteoid osteoma is a benign osteogenic tumor first reported by Jaffe in 1935 [1]. It is characterized by pain especially during the night, which is alleviated by nonsteroidal anti-inflammatory drugs. The excess production of prostaglandin in the nidus and abundance of nerve fibers have been postulated for the explanation of pain in osteoid osteoma [2]. Pain sometimes precedes the radiological identification of the lesion and may reside in an adjacent joint. These discrepancies between the clinical symptoms and radiologic findings often make a correct and timely diagnosis difficult. However, differential diagnosis of an osteoid osteoma is rather straightforward and should be always kept in mind for an early diagnosis and treatment in case of pain from an unknown cause.

Occurrence of an osteoid osteoma in the spinal column is rare; the incidence has been reported to be approximately 13 percent of all osteoid osteomas [3]. Osteoid osteoma arising in the sacrum is even rarer and only 18 cases have been reported to date in the English-language literature (Table 1) [4-9]. When an osteoid osteoma arises in the spinal column, it is usually in the posterior portion of the vertebrae. But, when the same tumor arises in the sacrum, 18 out of 19 cases, including our own case, arose in the anterior or lateral portion, and only one case occurred in the posterior articular process. This may be because the unique shape of the sacrum is different from other vertebra, but nevertheless it may aid in identification of the lesion when undergoing various imaging diagnoses.

**Table 1:** Past reports of osteoid osteoma of the sacrum

**Year**	**Authors**	**Time until diagnosis**	**Sacrum**	**Treatment**
1954	Cameron *et al.*[4]	12 months	1 case	Open resection
1986	Capanna *et al.*[5]	4-48 months	4 cases	Open resection
2001	Biagini *et al.*[6]	20.8 months	10 cases	Open resection
2002	Ozaki *et al.*[7]	NA	1 case	Open resection
2007	Barsa *et al.*[8]	NA	1 case	CT-guided resection
2009	Peyser *et al.*[9]	11 months	1 case	CT-guided resection
2013	Current study	12 months	1 case	CT-guided resection

CT, computed tomography; NA, not available.

Most of the osteoid osteomas that occurred in the sacrum were treated by open resection and only four cases were treated by CT-guided resection. CT-guided resection of the nidus was first reported by Voto *et al.* in 1990 [10]. Several percutaneous methods using CT guidance such as drilling, ethanol injection, and radiofrequency ablation have been reported, all yielding satisfactory results. Although it is less invasive compared to the conventional resection of the nidus, it has its shortcomings in that it is sometimes difficult to obtain enough tissue for a pathological diagnosis. Furthermore, recurrence rates have been reported to be up to 16 percent due to an incomplete resection or ablation of the tumor tissue [11,12]. However, in our experience, of the 18 cases treated by CT-guided resection in the past two decades, there was no local recurrence and histological diagnosis was achieved in 72 percent of the patients. When performing ablation near vital organs such as the spinal cord or nerve root, one must be aware of the heat conductivity inside the bone and CT-guided resection maybe compromised by the possible side effects. It has been reported that when treating osteoid osteoma, 90°C of heat should be applied for approximately five minutes for the necrosis of the entire tumor [9,13]. When this energy is applied to the tumor, a 2 to 5mm margin from the nerve has been postulated to be the safety threshold [14,15]. Although epidural vein plexus and cerebrospinal fluid may reduce the possible side effects, when the tumor is within 5mm of the spinal canal, it is prudent to undergo an open resection of the nidus.

## Conclusions

Here, we describe an osteoid osteoma of the sacrum treated by CT-guided resection. The present report shows that osteoid osteoma arising in the sacrum can successfully be treated by CT-guided resection without any noticeable side effects or local recurrence. Osteoid osteoma is sometimes difficult to detect especially in the early stage where there is no apparent radiographic findings. It is important to always bear in mind the possibility of osteoid osteoma for persisting pain, and to perform a thorough CT scan whenever the tumor is suspected.

## Consent

Written informed consent was obtained from the patient for publication of this case report and any accompanying images. A copy of the written consent is available for review by the Editor-in-Chief of this journal.

## Abbreviations

CT: computed tomography; MRI: magnetic resonance imaging; SPIR: selective partial inversion recovery.

## Competing interests

The authors declare that they have no competing interests.

## Authors’ contributions

SF carried out the data analysis and manuscript writing. MS completed the draft and critically revised the case report. IW and KN carried out the collection and assembly of data. KH contributed to the manuscript writing. YT provided administrative support. HM was responsible for the provision of study material of patients and final approval of the manuscript. All authors read and approved the final manuscript.
